# Severe Asthma in School-Age Children: An Updated Appraisal on Biological Options and Challenges in This Age Group

**DOI:** 10.3390/children12020167

**Published:** 2025-01-29

**Authors:** Cristiana Indolfi, Angela Klain, Maria Cristina Capuano, Simone Colosimo, Renata Rapillo, Michele Miraglia del Giudice

**Affiliations:** Department of Woman, Child and General and Specialized Surgery, University of Campania ‘Luigi Vanvitelli’, 80138 Naples, Italy; cristianaind@hotmail.com (C.I.); klainangela95@gmail.com (A.K.); simone.co345@gmail.com (S.C.); renata.rapillo1@gmail.com (R.R.); michele.miragliadelgiudice@unicampania.it (M.M.d.G.)

**Keywords:** severe asthma, children, school-age, treatment, biologicals, biomarkers

## Abstract

This review examines the growing role of biological therapies in managing severe asthma in children aged 6–11 years. Severe asthma, characterized by persistent symptoms and frequent exacerbations, presents significant challenges in pediatric care. Biologic treatments, including mepolizumab, omalizumab, and dupilumab, provide targeted interventions for patients with high eosinophilic inflammation or allergic asthma (T2-high asthma). Alongside their therapeutic benefits, the review evaluates the safety profiles of these biologics, highlighting potential side effects and the necessity for monitoring during long-term use. Cost considerations and treatment adherence also emerge as important challenges that need to be addressed in clinical practice. Additionally, the review emphasizes the need for identifying patients who would derive the most benefit from biologic therapies, advocating for the development of biomarkers to aid in treatment decisions. Emerging biologics, such as tezepelumab, are introduced as promising alternatives with the potential to target upstream inflammatory pathways, offering hope for treating T2-low asthma forms, which currently lack effective treatment options in children.

## 1. Introduction

Asthma is a widespread condition with substantial global health implications, affecting an estimated 300 million people worldwide. By 2025, this condition is expected to reach 400 million, driven by factors such as urbanization, environmental pollution, atopy, and lifestyle changes [1,2,3]. Asthma is a pathological condition characterized by chronic airway inflammation, which progressively leads to structural damage and remodeling of the bronchial wall. These changes affect the entire bronchial tree and contribute to the severity of clinical symptoms. A hallmark of asthma is bronchial hyperreactivity, defined as heightened airway sensitivity to various stimuli. This hyperreactivity is driven by the hypercontractility of bronchial smooth muscle, resulting in bronchoconstriction in response to even mild or non-specific triggers. Additionally, inflammatory mediators and epithelial damage further exacerbate airway responsiveness [4]. Asthma is associated with a higher prevalence and incidence in the pediatric population, affecting approximately 6.5% of school-age children, typically between 6 and 11 years of age [5]. The pharmacological approach to asthma therapy depends on the frequency and severity of symptoms and comorbid conditions [6]. The majority of pediatric patients can achieve good clinical control and maintain effective symptom management with low- or medium-dose inhaled corticosteroids (ICSs) as standard therapy [6]. Only 3% of school-age children have severe asthma that remains uncontrolled, with persistent symptoms and recurrent exacerbations despite maximal therapy or in which asthma control deteriorates upon step-down/tapering of therapy [4,7]. The anatomical and physiological features of pediatric airways make children more vulnerable to developing respiratory symptoms. Pediatric airways are more elastic, exhibit increased bronchial reactivity, and have a smaller diameter compared to those of adults, which amplifies the risk of bronchospasm and respiratory distress. The larynx is higher in infants, funnel-shaped, and narrowest at the cricoid cartilage. The trachea and bronchi in children are shorter, narrower, and more elastic, with growth in length and diameter occurring in phases during childhood and adolescence. Additionally, the chest wall in infants is highly compliant, with horizontal ribs and underdeveloped respiratory muscles. These features limit efficient ventilation and increase the risk of respiratory distress [8]. In children, the immune system is immature and still developing, which has significant implications for asthma pathophysiology. Mucociliary clearance and airway epithelial cell function are less efficient than in adults, rendering children more prone to respiratory infections. Newborns and young children exhibit a predominance of Th2 immune responses, which fosters allergic reactions and heightens the risk of asthma development. The thymus, highly active in early life, contributes to the generation of naive T lymphocytes; however, their antigen response is less robust compared to adults [9]. A deeper understanding of these age-specific immune and anatomical features is critical to identifying mechanisms driving asthma in children and informing the development of targeted biological therapies.

### Managing Severe Asthma in School-Age Children: Key Challenges

Although only about 3% of children aged 6–11 years have severe asthma, it is essential to consider issues related to treatment adherence and the accuracy of asthma diagnosis. Severe asthma in this age group requires careful evaluation to confirm the diagnosis and rule out other conditions, as well as addressing factors that may contribute to poor control. These factors include incorrect inhaler technique, inadequate adherence to therapy, associated comorbidities such as rhinosinusitis, gastroesophageal reflux disease (GERD), obesity, and obstructive sleep apnea (OSA), as well as persistent exposure to irritants like smoking or allergens [4]. Therefore, before addressing uncontrolled severe asthma, it is essential to reassess the patient’s inhaler technique, conduct a thorough review of their medical history, and rule out any comorbidities or contributing factors. Ensuring consistent adherence to prescribed therapies is critical, as non-adherence is a common issue that can lead to suboptimal outcomes, including increased exacerbations and impaired quality of life. Moreover, uncontrolled severe asthma during childhood can have long-term effects on lung development and function. When asthma is inadequately managed, children may experience chronic airway inflammation that compromises lung function. This can manifest as frequent episodes of breathing difficulties, acute asthma attacks, and an increased need for hospitalization, both for acute crisis management and long-term disease control. The condition can significantly impact the quality of life, limiting daily activities such as participation in sports, school, and social interactions, and leading to anxiety and depression. Over time, if severe asthma is not controlled, damage to the airways can become irreversible, resulting in permanent reductions in lung function and increasing the risk of developing chronic respiratory diseases, which should be considered based on the child’s asthma phenotype. In addition to pharmacological treatments, addressing comorbidities like allergic rhinitis and obesity is important, as is addressing obstructive pulmonary disease (COPD) in adulthood [10].

The Global Strategy for Asthma Management and Prevention (GINA) 2024 recommendations for pediatric patients aged 6–11 years with severe asthma recommend a personalized and progressive approach to achieve optimal control of symptoms, reduce exacerbations, and improve overall quality of life [4]. Severe asthma in this age group is characterized by persistent symptoms or frequent exacerbations despite high-dose ICSs combined with additional therapies [4]. Diagnosing severe asthma requires confirming the diagnosis through objective tests, such as spirometry or peak expiratory flow (PEF), and ruling out alternative conditions like vocal cord dysfunction or chronic infections [4]. Treatment follows a stepwise approach. Initial management includes high-dose ICSs combined with a long-acting beta2-agonist (LABA) and/or a leukotriene receptor antagonist. For children whose asthma remains uncontrolled, which corresponds to step 5, advanced therapies like biologics targeting specific inflammatory pathways are indicated to manage symptoms and avoid asthma triggers [10].

**Figure 1:**
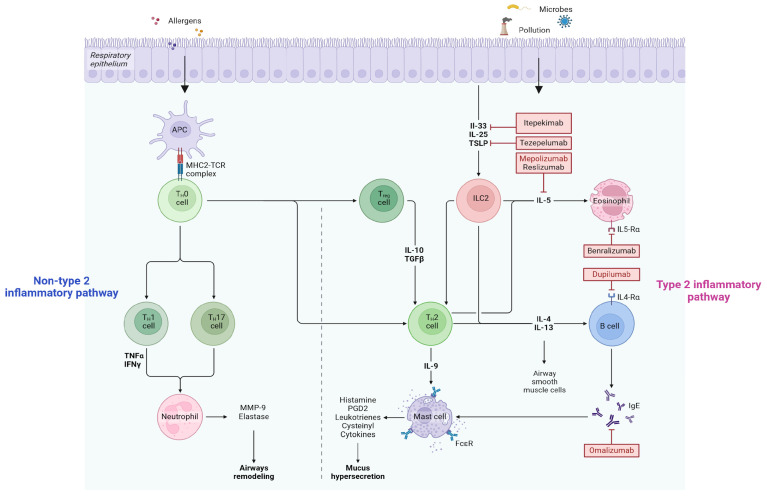
The image illustrates the inflammatory pathways in asthma, differentiating between type 2 and non-type 2 responses. In the type 2 pathway, epithelial cells, in response to allergens or environmental factors (e.g., microbes or pollutants), release alarmins such as IL-33, IL-25, and TSLP, which act upstream to activate ILC2 and Th2 cells. This triggers the production of IL-4, IL-5, and IL-13, leading to eosinophil activation, B-cell-mediated IgE production, mast cell activation, mucus hypersecretion, and airway smooth muscle contraction. Drugs like itepekimab, tezepelumab, mepolizumab, reslizumab, benralizumab, dupilumab, and omalizumab target specific mediators in this pathway. The non-type 2 pathway involves neutrophils driven by Th1 and Th17 cells via TNFα and IFNγ, contributing to airway remodeling through MMP-9 and elastase. Regulatory T cells (Tregs) modulate inflammation by releasing IL-10 and TGFβ. Approved biological therapies for children aged 6–11 years are highlighted in red. Figure created in https://BioRender.com (accessed on 24 January 2025), adapted from “Asthma Treatments: Inflammation Pathways, Biomarkers, and Mechanisms of Action, by BioRender.com (2024). Retrieved from https://app.biorender.com/biorender-templates (accessed on 24 January 2025).

Biological therapies enable the personalization of asthma treatment by targeting specific asthma phenotypes, thereby advancing the field of precision medicine and targeted therapy [11]. Asthma in children is predominantly driven by Th2-type inflammation, characterized by the activation of T-helper 2 (Th2) cells and the overproduction of cytokines such as IL-4, IL-5, and IL-13. These cytokines play a central role in promoting IgE production, eosinophilic inflammation, and airway hyperresponsiveness [12]. In pediatric asthma, this mechanism is often associated with allergic sensitizations, making it the most common phenotype in this age group, the T2-high phenotype. The presence of high serum IgE levels, eosinophilia, high fractional exhaled nitric oxide (FeNO), and responsiveness to corticosteroids are hallmark features of the T2-high phenotype. The approved biological drugs, mepolizumab, dupilumab, and omalizumab target specific mediators in this pathway. Although less common, the T2-low phenotype in children may present unique clinical features and challenges, including reduced responsiveness to standard therapies targeting T2 inflammation. T2-low inflammation is characterized by neutrophilic or paucigranulocytic inflammation, low FeNO, normal or low IgE, and poor responsiveness to corticosteroids. Key markers include elevated IL-8, IL-6, and IL-17, with reduced T2 cytokines (IL-4, IL-5, IL-13), highlighting the need for alternative therapeutic approaches. Epithelial–mesenchymal interactions are another crucial mechanism contributing to airway remodeling in pediatric asthma. Airway epithelial cells, in response to environmental allergens or pollutants, release alarmins such as IL-33, thymic stromal lymphopoietin (TSLP), and IL-25. These alarmins activate innate and adaptive immune pathways, including Th2 cells and type 2 innate lymphoid cells (ILC2s), amplifying inflammation. Additionally, epithelial damage enhances the crosstalk with mesenchymal cells, such as fibroblasts and myofibroblasts, promoting extracellular matrix deposition and subepithelial fibrosis. Novel therapies targeting alarmins, such as anti-IL-33 (e.g., itepekimab) or anti-TSLP (e.g., tezepelumab), which act upstream of the T2-high and T2-low pathways, are emerging as promising strategies for disrupting these pathological pathways (Figure 1).

Recently, tezepelumab has been approved for the treatment of severe asthma in adolescents. This biological therapy targets TSLP, a key cytokine involved in the inflammatory pathways of asthma [13]. Acting upstream in the Th2 cascade, it can also be effective in T2-low-mediated forms of asthma, broadening its potential applicability for patients with severe asthma who do not exhibit the typical type 2 inflammatory profile.

Biological therapies not only improve clinical symptoms and reduce the frequency of exacerbations, but also contribute to a significant reduction in asthma-related morbidity, mortality, and healthcare costs. As a result, it becomes possible to use lower doses of maintenance medications and rescue medications, such as short-acting β2-agonists and oral corticosteroids [14,15].

In this review, we have outlined the current treatment options for school-age children with uncontrolled severe asthma, highlighting all the challenges and opportunities in this age group.

## 2. Approved Biological Therapies in School-Age Children

### 2.1. Dupilumab

Dupilumab is a fully human monoclonal antibody designed to target the interleukin-4 receptor alpha (IL-4Rα) subunit, thereby blocking the signaling pathways of IL-4 and IL-13, key drivers of Th2 inflammation. It is approved for the therapy of moderate-to-severe asthma, particularly in patients with a T2 inflammatory phenotype, including elevated blood eosinophils or fractional exhaled nitric oxide (FeNO) [16,17,18]. Dupilumab is also indicated for other type 2 inflammatory conditions, such as atopic dermatitis (AD) and chronic rhinosinusitis with nasal polyps. It is administered via subcutaneous injection. The randomized, double-blind controlled trial VOYAGE investigated the efficacy and safety of dupilumab in children aged 6 to 11 years with moderate/severe asthma. This study led to its approval for this age group first in the United States by the Food and Drug Administration (FDA) in 2021 [19], then by the European Medicines Agency (EMA) in Europe in 2022 [20], and in Italy by Agenzia Italiana Del Farmaco (AIFA) in 2024 [21].

The dosing of dupilumab is based on body weight. Children weighing 15 to less than 30 kg receive 300 mg every four weeks (Q4W). Children weighing 30 kg to less than 60 kg can receive either 200 mg every other week (Q2W) or 300 mg every four weeks (Q4W). Children weighing 60 kg or more receive 200 mg every other week (Q2W) [22].

The VOYAGE study involved 408 children aged 6–11 with uncontrolled moderate-to-severe asthma, randomized to receive either dupilumab (*n* = 273) or placebo (*n* = 135) alongside standard therapy for 52 weeks. In the primary analysis, dupilumab reduced the annualized severe exacerbation rate to 0.31 compared to 0.75 for placebo, a 59.3% reduction (*p* < 0.001). In those with eosinophils ≥ 300/μL, the reduction was greater, 64.7% (*p* < 0.001) [23]. Dupilumab improved prebronchodilator FEV1 by 10.5 ± 1.0 percentage points compared to 5.3 ± 1.4 percentage points with placebo at week 12, a mean difference of 5.2 points (*p* < 0.001). Moreover, dupilumab significantly reduced the Asthma Control Questionnaire (ACQ-7) in the treated group compared to placebo (*p* < 0.001). Adverse events occurred in 83% of dupilumab-treated and 79.9% of placebo-treated patients, with no significant differences in serious adverse events. The most common adverse event that occurred was viral infection of the upper respiratory tract. Moreover, dupilumab significantly reduced FeNO levels compared to placebo (*p* < 0.001), too [23,24,25].

In children receiving dupilumab 100 mg every 2 weeks and 200 mg every 2 weeks, reductions in type 2 biomarkers (serum total immunoglobulin E, thymus and activation-regulated chemokine, blood eosinophils, fractional exhaled nitric oxide) were similar [26].

In addition, the efficacy of dupilumab in reducing exacerbations and improving lung function has been demonstrated to be independent of baseline ICS dose, whether medium or high [27], and unaffected by baseline patient demographic or disease characteristics [28] or by evidence of allergic asthma [29].

A subsequent study, conducted as part of the VOYAGE trial, investigated the effects of dupilumab on various spirometric parameters, extending beyond just FEV1. Prebronchodilator FEF25–75% improved by 0.30 l (*p* < 0.001), reflecting enhanced small airway function. These improvements were observed as early as week 2 and sustained through week 52, with greater effects in children with elevated biomarkers of type 2 inflammation (eosinophils ≥ 150 cells/μL or FeNO ≥ 20 ppb). Dupilumab also reduced the proportion of children with postbronchodilator airflow obstruction (FEV1/FVC Z-score < −1.64) from 33.3% to 20.6% (*p* = 0.04) [25,30].

The LIBERTY asthma EXCURSION study, an open-label extension study, followed pediatric patients who completed the VOYAGE study, to assess long-term outcomes. Over 96 weeks, dupilumab maintained reductions in severe exacerbations and sustained improvements in lung function, with no new safety concerns emerging, reaffirming its tolerability for prolonged use [31,32].

A recent study by Piacentini et al. focused on identifying pediatric patients in Italy eligible for treatment with dupilumab for Th2 asthma. This biomarker-based analysis employed a two-phase method to assess the population. It began by estimating the number of children aged 6–11 years with uncontrolled severe asthma and then stratified them based on elevated biomarker levels, such as eosinophils, FeNO, and IgE. According to the findings, 1007 Italian children aged 6–11 years had uncontrolled severe asthma; the majority (about 74%) of patients had at least two elevated biomarkers, and more than 85% of the patients were considered eligible for dupilumab treatment [33].

The use of dupilumab in pediatric patients presents a promising opportunity to alter the course of allergic diseases, particularly in the context of the atopic march. The findings from clinical studies, including those by Geba et al., suggest that dupilumab can help mitigate the progression of atopic comorbidities, particularly when administered in younger patients with early-onset and severe forms of allergic conditions like atopic dermatitis (AD) [34]. This is especially important as the T2 immune response, which plays a key role in allergic diseases, seems to remain modifiable even into adolescence, highlighting a critical window for intervention. Early treatment with dupilumab could potentially reduce the incidence of new allergies and prevent the development of more severe allergic comorbidities, such as asthma, over time. Although the current understanding is that no single treatment works universally across all patients, the precision medicine approach—guided by biomarkers and tailored to individual responses—could significantly improve treatment outcomes. Early intervention with dupilumab, especially in children with severe or persistent allergic conditions, could therefore be a game changer in halting or even modifying the atopic march [35].

### 2.2. Mepolizumab

Mepolizumab is a monoclonal antibody that blocks interleukin-5 (IL-5) by binding to it and inhibiting its interaction with the IL-5 α receptor. IL-5 is a key cytokine in eosinophils’ maturation, activation, and survival. Through the inhibition of IL-5, mepolizumab reduces the eosinophil count in the blood and tissues, contributing to better asthma control and reducing the risk of exacerbations [36,37].

Mepolizumab received approval for use in children with severe eosinophilic asthma starting from the age of 6 in the United States in 2019 [38]. In Europe, it has been available for the treatment of severe eosinophilic asthma in children aged 6 years and older since August 2018. The key criteria for mepolizumab administration include a confirmed diagnosis of severe eosinophilic asthma, supported by markers such as elevated FeNO levels; a blood eosinophil count exceeding 150 cells/µL at the time of assessment or over 300 cells/µL in the past 12 months; and persistent symptoms that remain uncontrolled despite high-dose inhaled corticosteroids combined with LABA [36,39].

The recommended dosage for the age group 6–11 years is 40 mg administered subcutaneously every four weeks for children weighing less than 40 kg, while for those weighing more, the dosage of 100 mg is used [40,41].

The approval of mepolizumab in children between 6 and 11 years of age is mainly based on safety data from a specific randomized-control trial (RCT) published in 2018 [42]. A total of 36 children were enrolled and stratified by body weight into two dosing groups: 40 mg for those under 40 kg and 100 mg for those 40 kg or above, with administration every four weeks over a 12-week period. Mepolizumab exposure levels were higher than predicted but remained within two-fold of adult levels, with bodyweight-adjusted exposures of 454 μg/day/mL and 675 μg/day/mL for the 40 mg and 100 mg groups, respectively. Blood eosinophil counts showed a significant reduction from baseline, decreasing by 88.5% in the 40 mg group and 83.4% in the 100 mg group, with eosinophil levels at week 12 reduced to 42 cells/μL and 55 cells/μL, respectively (*p* < 0.001 for both groups). Improvements in asthma control were indicated by changes in the ACQ-7 and Childhood Asthma Control Test (C-ACT), with 48% of participants achieving a clinically meaningful improvement in ACQ-7 scores by week 12. However, no significant changes in lung function, as measured by FEV1, were observed during the study period. Mepolizumab was well tolerated, with adverse events reported in 72% of participants, most of which were mild or moderate, including headache and injection site reactions. Serious adverse events occurred in 19% of children, though only 6% were considered related to the study drug, and no new safety concerns emerged compared to prior studies in older populations [42,43]. Additionally, a recent study involving 514 patients, including 24 aged 6–17 years, confirmed the safety of the treatment for up to 10 years of use, with a positive risk–benefit balance even in pediatric patients [40].

The trial by Jackson et al. evaluated the efficacy and safety of mepolizumab in 290 urban children and adolescents (aged 6–17 years) with exacerbation-prone eosinophilic asthma. Participants were randomly assigned to receive mepolizumab (146 patients; 40 mg for children aged 6–11 years, 100 mg for adolescents aged 12–17 years) or placebo (144 patients) every 4 weeks for 52 weeks. The primary outcome was the rate of asthma exacerbations treated with systemic corticosteroids. Mepolizumab significantly reduced the annualized asthma exacerbation rate to 0.96 (95% CI, 0.78–1.17) compared to 1.30 (95% CI, 1.08–1.57) in the placebo group, with a rate ratio of 0.73 (95% CI, 0.56–0.96; *p* = 0.027). Seasonal patterns of exacerbations were also modulated, with a significant reduction in the fall exacerbation peak (*p* = 0.041). However, there were no significant differences between groups in secondary outcomes, including time to first exacerbation (HR 0.86; 95% CI, 0.63–1.18; *p* = 0.36), lung function measures, or patient-reported asthma severity indices. Mechanistic analyses revealed that mepolizumab reduced eosinophil-associated inflammatory pathways, such as T2 inflammation, eicosanoid metabolism, and eosinophil cytoplasmic proteins, which were associated with higher exacerbation risk in the placebo group (*p*-values ranging from 0.0012 to 0.015). In contrast, epithelial inflammatory pathways (e.g., keratinization, extracellular matrix production, and IL-33 response) were significantly upregulated in the mepolizumab group and correlated with persistent exacerbation risk (*p*-values ranging from 0.0035 to 0.030). Mepolizumab was generally well tolerated, though injection site reactions were more frequent in the treatment group (13% vs. 4.9% with placebo) [44].

A recent study examined the use of mepolizumab, an anti-IL-5 monoclonal antibody, in children and adolescents with severe eosinophilic asthma (SA) through a case series of four pediatric patients, aged 10 to 16 years. After 12 months of mepolizumab therapy, blood eosinophil counts were significantly reduced by 81–93% (*p* < 0.001), asthma exacerbations decreased by over 50% in frequency, and ACT scores improved across all cases, with clinically significant increases of 6–7 points (*p* < 0.05). Spirometry results, including FEV1, showed no substantial changes pre- and post-treatment [45].

The study by Wilson et al. investigated the factors associated with the persistence of airway eosinophils in children with severe asthma treated with mepolizumab. Sputum eosinophils were analyzed in 53 children aged 10 years or older using advanced mass cytometry techniques. The analysis identified three distinct eosinophil subpopulations differentiated by CD62L expression: CD62Llo, CD62Lint, and CD62Lhi. While mepolizumab-treated participants showed a significant reduction in overall sputum eosinophil levels compared to placebo (58% lower; *p* = 0.04), subpopulations CD62Lint and CD62Lhi were significantly elevated in those who experienced exacerbations despite treatment (100% higher for CD62Lint, *p* = 0.04; 93% higher for CD62Lhi, *p* = 0.04). These subpopulations displayed unique functional profiles, including higher levels of activation markers, inflammatory mediators, and eosinophil peroxidase expression. These findings suggest that CD62Lint and CD62Lhi eosinophils contribute to breakthrough exacerbations via IL-5-independent pathways, potentially involving IL-4 signaling [46].

### 2.3. Omalizumab

Omalizumab is the first biologic drug approved for the treatment of moderate-to-severe allergic asthma, receiving approval in 2003 [47]. It is a humanized monoclonal antibody targeting IgE, specifically binding to the same site as the high-affinity IgE receptor (FcεRI). By attaching to IgE, it blocks IgE’s interaction with antigen-presenting cells, mast cells, and basophils. This action decreases the level of cell-bound IgE, downregulates high-affinity IgE receptors, and inhibits the release of mediators from effector cells. Furthermore, it helps to prevent inflammatory responses, tissue remodeling, and chronic Th2-driven inflammation [48,49]. Recent research supports its potential antiviral role: it blocks the interaction of IgE with membrane receptors on plasmacytoid dendritic cells (pDCs), thereby preventing virus binding and enhancing the release of interferon-α, which stimulates the activation of the innate immune response [50]. This evidence is particularly important in pediatric patients, especially those with asthma, as they are more likely to experience frequent respiratory infections.

Licensed for the treatment of severe asthma in adults and adolescents since 2003 by the FDA and 2005 by the EMA, omalizumab has been approved for children aged 6–11 years since 2009 by the EMA [51]. From that year until 2019, it was the only biological drug approved in Europe as an add-on therapy for children aged ≥ 6 years with severe allergic asthma that remained uncontrolled despite high-dose ICS and LABA treatment. Eligibility required positive allergy testing and IgE levels of 30–1300 IU/mL (for children aged 6–11 years in the United States), 30–700 IU/mL (for individuals aged ≥ 12 years in the United States), or 30–1500 IU/mL (in the European Union) [52]. In Italy, omalizumab was approved for use in children aged 6–11 years in 2014 [53]. The injection dosage is determined based on IgE concentration and body weight (0.016 mg/kg per IU of IgE over a 4-week period), with subcutaneous administration every 2–4 weeks (150–375 mg in the United States and 150–600 mg in the European Union). The FDA identifies the most common adverse events associated with omalizumab for asthma as nasopharyngitis, headache, fever, upper abdominal pain, streptococcal pharyngitis, otitis media, viral gastroenteritis, insect bites, and nosebleeds [54].

In 2001, Milgrom et al. conducted a landmark RCT that highlighted the potential of omalizumab in improving asthma management for children aged 6–12 years with moderate/severe asthma. This double-blind, placebo-controlled trial spanned 28 weeks and included 334 children (225 in the active group and 109 in the placebo group) on stable ICS therapy with beclomethasone dipropionate. Patients treated with omalizumab achieved a median 100% reduction in beclomethasone dipropionate (BDP) doses compared to 66.7% in the placebo group (*p* = 0.001), with 55% discontinuing BDP entirely versus 39% in the placebo group (*p* = 0.004). Asthma exacerbations requiring treatment were significantly lower in the omalizumab group during the steroid-reduction phase, with 18.2% experiencing exacerbations compared to 38.5% in the placebo group (*p* < 0.001) and a mean of 0.42 episodes per patient versus 2.72 in the placebo group (*p* < 0.001). No exacerbations requiring hospitalization occurred in the omalizumab group, whereas five such events were observed in the placebo group. Rescue medication use was consistently lower in the omalizumab group, with a statistically significant reduction by week 28 (*p* = 0.004). Omalizumab effectively reduced serum-free IgE levels by 95–99% while increasing total IgE due to inactive complexes, demonstrating its targeted pharmacological action. The treatment was well tolerated, with no serious drug-related adverse events reported. Mild-to-moderate urticaria was observed more frequently in the omalizumab group (4% vs. 0.9%, *p* = 0.029) [55]. These findings have also been confirmed in other RCTs [56,57,58,59], with evidence of reducing FeNO levels [60] and improving asthma-related quality of life (AQoL) [61,62]. Observational studies, cohort studies, and case series have also demonstrated the effectiveness of omalizumab in reducing exacerbations and the use of oral corticosteroids, while maintaining a notable safety profile [50,63,64,65,66].

Omalizumab is also approved for chronic idiopathic urticaria that is symptomatic despite H1 antihistamine treatment [54].

In February 2024, omalizumab was licensed for food allergy by the FDA. Omalizumab is now indicated for IgE-mediated FA in adults and children aged 1 year or older to reduce allergic reactions [67,68].

While not yet widely licensed elsewhere, it is gaining recognition as a potential off-label option. Current evidence supports its use as monotherapy in specific cases, such as patients with recurrent severe anaphylaxis or those requiring short-term risk reduction (e.g., due to travel or high-risk occupations). Omalizumab may also serve as a bridge to oral immunotherapy or food introduction, potentially improving tolerance thresholds [69]. The recent study by Arasi et al. examined the impact of omalizumab on children aged 6–18 years with severe asthma and food allergies. Over 12 months, 65 participants underwent oral food challenges (OFCs) at 4-month intervals to assess tolerance thresholds for various allergens. At the first assessment, 66.4% of foods were tolerated, increasing to 75% by the third, enabling 40 participants to achieve full dietary liberalization. Median increases in the no observed adverse events level (NOAEL) ranged from 243-fold for milk to 10-fold for fish. Anaphylactic episodes decreased significantly from 39 to 7 annually. ACT scores improved from 17 to 23.6, and quality-of-life scores showed marked reductions in limitations for children and adolescents. Omalizumab demonstrated efficacy in reducing allergic reactions, improving tolerance, and enhancing quality of life while being well tolerated [70]. These findings support its use as a promising therapeutic option in complex cases involving comorbid severe asthma and food allergies.

Table 1 summarizes the characteristics of approved biologic drugs for school-age children (Table 1).

## 3. Discussion

**Figure 2:**
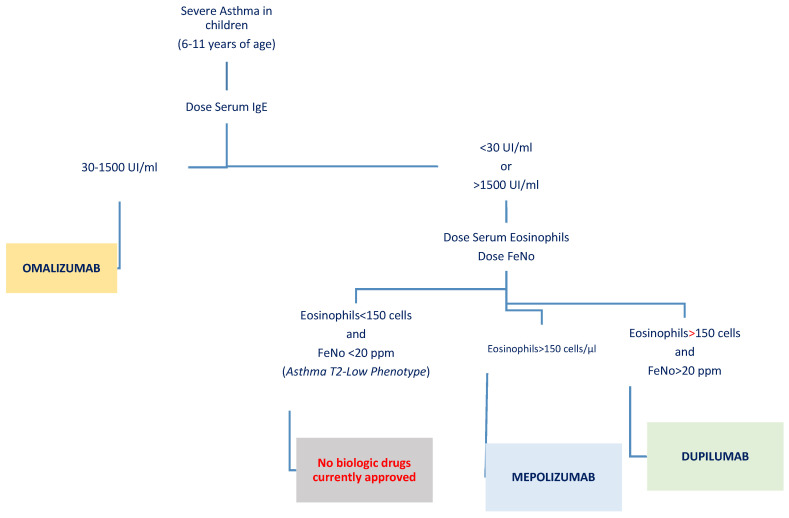
Flowchart illustrating the selection of the most appropriate biological therapy for children aged 6–11 years with severe asthma, based on available biomarkers.

Currently approved biologics in children aged 6–11 years, including omalizumab, mepolizumab, and dupilumab, target Th2 inflammation through a blockade at various levels, reduce severe exacerbations, and improve asthma control. The main considerations for the use of biologic therapy in pediatric patients with severe asthma involve several critical factors that must be carefully addressed:

- Adherence to treatment: Pediatric patients often face unique challenges regarding compliance with biologic therapies, which are typically injectable. Younger children might find the injections uncomfortable or distressing, and their understanding of the importance of consistent treatment may be limited [71]. This highlights the need for strong caregiver involvement and educational strategies to support adherence and ensure the therapy is administered correctly and consistently.
- Safety considerations: Although the safety profiles of these biological therapies are generally reassuring, offering a strong foundation for their use, with serious side effects being rare, safety remains a primary concern in younger populations. In children, the risk of adverse effects must be continuously monitored, as their developing immune systems and unique physiology may react differently to treatment. Vigilance in identifying and managing even rare side effects, such as injection site reactions, is particularly important in this age group.
- Duration of therapy and discontinuation: A significant challenge in pediatric biologic therapy is determining the appropriate length of treatment. Long-term data in children are limited, and questions remain about the optimal timing for discontinuation. Clinicians need to consider what happens when therapy is stopped—whether symptoms will rebound, if disease control will deteriorate, or if treatment can be tapered without compromising outcomes. The existing data on omalizumab show conflicting results [72]. Addressing these questions requires more robust data from longitudinal studies targeting pediatric populations [73].
- Identification of biomarkers: Choosing the right biological therapy for each patient requires a detailed understanding of their disease phenotype and the use of reliable biomarkers (Figure 2). Personalized treatment strategies, which take into account individual biomarkers and comorbid conditions, can significantly enhance the likelihood of achieving optimal therapeutic outcomes. Yet, several challenges persist, including the lack of robust head-to-head studies comparing these biologics and the fact that responses to treatment can vary widely across patients. While markers for identifying the T2-high phenotype are well established, IL-4, IL-5, IL-13, IgE, eosinophilia, and high FeNO levels, evidence for T2-low biomarkers remains limited [74,75].

Sputum induction (SI) represents the gold standard for assessing bronchial inflammation in asthma patients, enabling differentiation among four inflammatory phenotypes: eosinophilic, paucigranulocytic, neutrophilic, and mixed [76].

- Impact on healthcare sustainability: A balance exists between the advantages and costs of biological therapies for children with severe asthma. These treatments markedly reduced hospital admissions, emergency room visits, and reliance on oral corticosteroids, enhancing asthma control and addressing comorbidities. However, they also significantly increased overall healthcare expenses. This trade-off highlights the importance of weighing clinical benefits against financial impacts and emphasizes the need for thoughtful, individualized use of biologics to ensure healthcare sustainability [77].
- The ‘modifying disease’ effect and prevention impact: Recent findings underscore the potential of biologics to not only manage symptoms of allergic diseases but also prevent their progression, a concept integral to the atopic march [35]. The atopic march describes the sequential development of allergic conditions, beginning with AD in infancy and advancing to food allergies, asthma, and allergic rhinitis in later childhood or adolescence. Dupilumab has shown promising results in reducing the onset of new allergic comorbidities in individuals with severe early-onset AD [78]. Meta-analyses of clinical trials suggest that early intervention with dupilumab significantly decreases the risk of additional allergic diseases by modulating the immune response during a critical window of immune system plasticity [34]. This preventative effect is particularly pronounced in younger patients, where the immune system remains more adaptable, allowing biologics to effectively recalibrate inflammatory pathways before the full establishment of chronic allergic patterns. For instance, reductions of up to 37% in the incidence of new allergies and even greater effects when targeting IgE-mediated conditions suggest the potential for profound impacts on long-term disease outcomes. Omalizumab has also broadened its indications to include severe food allergies, marking a critical step forward in the prevention of severe reactions [79,80]. This effect not only lowers the risk of life-threatening reactions but also alleviates the psychological burden of food allergies, improving the quality of life of children and their families [81]. The ability of omalizumab to modulate immune responses highlights its role in potentially preventing the progression of allergic conditions by reducing the severity and frequency of sensitizations over time. The concept of prevention through biologics extends beyond individual conditions to the broader atopic march. However, achieving this preventative potential requires a better understanding of optimal intervention windows and patient selection criteria [82]. Current data suggest that biologics offer a promising avenue for reshaping the management of allergic diseases, not only by controlling symptoms but by fundamentally altering the immune landscape to reduce the development of future allergies. Further research is essential to refine these strategies, ensuring biologics achieve their full potential as preventative tools while balancing efficacy, cost, and long-term sustainability.

## 4. Conclusions

Biological therapies demonstrate the ability to enhance lung function, following distinct trajectories across different treatments, highlighting variability in patient responses. This highlights the need for tailored treatments for individual patients and the development of novel biomarkers to manage non-responders or those with distinct asthma phenotypes [83]. Future perspectives for severe asthma in pediatric patients also include the use of new drugs with novel therapeutic targets, such as tezepelumab and benralizumab.

Tezepelumab is a human IgG2 monoclonal antibody that targets thymic stromal lymphopoietin (TSLP), a key regulator of airway inflammation. TSLP, produced by airway epithelium and immune cells, triggers signaling pathways that drive Th2 responses, leading to the release of IL-4, IL-5, and IL-13 [14,84]. Due to its mechanism of action, which targets upstream inflammatory pathways, tezepelumab is currently the only option for T2-low or non-T2-mediated asthma in patients who are not eligible for other available biologics. Tezepelumab, approved for treating severe asthma in patients aged ≥ 12 years from NAVIGATOR study results [85], is evaluated in the ongoing TRAILHEAD study, assessing pharmacokinetics (PK), pharmacodynamics (PD), and safety in children aged 5–11 years with asthma. In this phase 1, multicenter, open-label trial, 18 children received a single subcutaneous 70 mg dose of tezepelumab. Preliminary results are consistent with prior studies, and the study continues to support the further development of tezepelumab for children with asthma [86].

Benralizumab is a monoclonal antibody targeting the alpha subunit of the interleukin-5 receptor (IL-5Rα) approved for the treatment of severe and uncontrolled asthma in patients aged ≥ 12 years. The TATE study, a phase 3 open-label trial, evaluated benralizumab in children aged 6–11 years with severe eosinophilic asthma. Participants were divided into two groups based on weight: one group received 10 mg (weight < 35 kg) and the other 30 mg (weight ≥ 35 kg). The results demonstrated that benralizumab was well tolerated, with near-complete eosinophil depletion in both groups. Participants in the higher-dose group showed the most significant improvements in pulmonary function (FEV1) and asthma control (ACQ-IA). Safety outcomes were consistent with previous studies in adults and adolescents, with no new safety signals identified [87].

Other potential therapeutic options in the future could include itepekimab, a human monoclonal antibody that inhibits IL-33, which is under consideration in adults with moderate-to-severe asthma [88], and inhibitors targeting Janus kinases (JAKs), enzymes crucial for the signal transduction of inflammatory cytokines, particularly those implicated in type 2 asthma [89] (Table 2).

## Figures and Tables

**Table 1:** Summary of approved biologic drugs for school-age children.

Biologic Therapy	Mechanism of Action	Indications	Dosage and Administration	Key Studies and Findings	Safety Profile
Dupilumab	Targets IL-4Rα to inhibit IL-4 and IL-13 signaling, reducing Th2 inflammation.	Moderate-to-severe asthma with T2 inflammation (elevated eosinophils or FeNO); also approved for AD and CRSwNP.	Subcutaneous injection based on weight: <30 kg: 300 mg Q4W; 30–60 kg: 200 mg Q2W or 300 mg Q4W; >60 kg: 200 mg Q2W.	VOYAGE study: 59.3% reduction in annual exacerbation rate; improved FEV1 (+5.2 points over placebo); reduced FeNO and ACQ-7 scores. Long-term efficacy and safety confirmed in LIBERTY Asthma EXCURSION. Early intervention may modify the atopic march.	Common: Viral URTIs, injection site reactions. Rare: Serious adverse events comparable to placebo.
Mepolizumab	Anti-IL-5 antibody inhibiting eosinophil maturation and survival.	Severe eosinophilic asthma with elevated FeNO or eosinophils (>150/µL at assessment or >300/µL in 12 months).	Subcutaneous injection every 4 weeks: <40 kg: 40 mg; ≥40 kg: 100 mg.	Gupta et al.’s study: Reduced annualized exacerbations (0.96 vs. 1.30 in placebo); significant eosinophil reduction (>80%); improvements in ACQ-7 and C-ACT scores but no significant FEV1 changes. Jackson et al.’s research: Reduced fall exacerbation peak; some pathways independent of IL-5 identified.	Common: Headache, injection site reactions. Rare: Serious adverse events in 6%, unrelated to treatment.
Omalizumab	Anti-IgE antibody reducing cell-bound IgE and downregulating IgE receptors.	Moderate-to-severe allergic asthma (positive allergy tests, IgE 30–1500 IU/mL); also licensed for chronic idiopathic urticaria and food allergies.	Dosed by IgE and weight: 0.016 mg/kg per IU of IgE/4 weeks; subcutaneous injection every 2–4 weeks (150–375 mg US; up to 600 mg EU).	Landmark and Milgrom RCT: 100% median reduction in ICS dose; 55% discontinued ICSs. Significant reduction in exacerbations (0.42 vs. 2.72 episodes). Arasi et al.: Improved food tolerance and reduced anaphylactic episodes in children with comorbid food allergies. Potential as an adjunct to oral immunotherapy.	Common: Headache, URTIs, injection site reactions. Rare: Anaphylaxis; patients should be monitored after administration.

**Table 2:** Overview of potentially approvable biologic drugs for school-age children.

Drug	Target	Main Indications	Studies and Results	Dosage	Side Effects	Approval Status
Tezepelumab	TSLP (Thymic Stromal Lymphopoietin)	Severe asthma with non-specifically T2 inflammation	PATHWAY and NAVIGATOR studies: significant reduction in asthma exacerbations (up to 71% in NAVIGATOR) regardless of type 2 biomarkers (eosinophils or FeNO). Improvements in lung function (FEV1), asthma control, and quality of life. Efficacy demonstrated even in patients with low levels of eosinophils or FeNO.	210 mg administered subcutaneously every 4 weeks.	Upper respiratory infections, injection site reactions, headache.	FDA-approved in 2021 for adults and adolescents; pediatric approval under review.
Benralizumab	IL-5Rα	Severe eosinophilic asthma	SIROCCO, CALIMA, and BORA studies: reduction in asthma exacerbations (up to 51–70%); reduction in eosinophil levels to nearly zero. Significant improvements in lung function and quality of life.	30 mg subcutaneous every 4 weeks for 3 doses, then every 8 weeks.	Injection site reactions, headache, upper respiratory tract viral infections.	FDA- and EMA-approved for patients aged >12; pediatric use under review.
Itepekimab	IL-33	Severe asthma (both eosinophilic and non-eosinophilic)	ECLIPSE study: significant reduction in asthma exacerbations in patients with high IL-33 levels. Improvements in lung function (FEV1) and symptoms. Effective both in the presence and absence of elevated T2 biomarkers (e.g., eosinophils).	Under study, variable dosages administered every 4 weeks.	Injection site reactions, fever, flu-like symptoms.	In clinical trial phase.

## Data Availability

No new data were created or analyzed in this study. Data sharing is not applicable to this article.

## Abbreviations

The following abbreviations are used in this manuscript:

ICS	Inhaled Corticosteroid
GERD	Gastroesophageal Reflux Disease
OSA	Obstructive Sleep Apnea
PEF	Peak Expiratory Flow
LABA	Long-Acting Beta2-Agonist
GINA	Global Initiative for Asthma
FeNO	Fractional Exhaled Nitric Oxide
T2	Type 2
IL	Interleukin
IgE	Immunoglobulin E
FDA	Food and Drug Administration
EMA	European Medicines Agency
AIFA	Agenzia Italiana Del Farmaco (Italian Medicines Agency)
Q4W	Every Four Weeks
Q2W	Every Two Weeks
ACQ-7	Asthma Control Questionnaire 7
FEV1	Forced Expiratory Volume in 1 Second
FVC	Forced Vital Capacity
EF25–75%	Forced Expiratory Flow 25–75%
Th2	T-helper 2 (Cells)
T2-high	Type 2 High (Phenotype)
T2-low	Type 2 Low (Phenotype)
IL-5	Interleukin-5
RCT	Randomized Controlled Trial
C-ACT	Childhood Asthma Control Test
OFC	Oral Food Challenge
NOAEL	No Observed Adverse Effect Level
BDP	Beclometasone Dipropionate
PK	Pharmacokinetics
PD	Pharmacodynamics
TSLP	Thymic Stromal Lymphopoietin
IL-13	Interleukin-13
JAK	Janus Kinase
